# Analysis of Population Structure and Genetic Diversity in Rice Germplasm Using SSR Markers: An Initiative Towards Association Mapping of Agronomic Traits in *Oryza Sativa*

**DOI:** 10.1186/s12284-015-0062-5

**Published:** 2015-09-26

**Authors:** Vishnu Varthini Nachimuthu, Raveendran Muthurajan, Sudhakar Duraialaguraja, Rajeswari Sivakami, Balaji Aravindhan Pandian, Govinthraj Ponniah, Karthika Gunasekaran, Manonmani Swaminathan, Suji K K, Robin Sabariappan

**Affiliations:** Plant Molecular Biology, Plant Breeding and Genetics Divison, International Rice Research Institute, Manila, Philippines; Centre for Plant Breeding and Genetics, Tamil Nadu Agricultural University, Coimbatore, India; Centre for Plant Molecular Biology and Biotechnology, Tamil Nadu Agricultural University, Coimbatore, India; Crop Physiology laboratory, International Crops Research Institute for the Semi-Arid-Tropics, Hyderabad, India; International Crops Research Institute for the Semi-Arid-Tropics, Hyderabad, India; Centre of Excellence in Molecular Breeding, Tamil Nadu Agricultural University, Coimbatore, India

**Keywords:** Rice, Genetic diversity, Population structure, Polymorphism information content, Molecular variance, Association mapping

## Abstract

**Background:**

Genetic diversity is the main source of variability in any crop improvement program. It serves as a reservoir for identifying superior alleles controlling key agronomic and quality traits through allele mining/association mapping. Association mapping based on LD (Linkage dis-equilibrium), non-random associations between causative loci and phenotype in natural population is highly useful in dissecting out genetic basis of complex traits. For any successful association mapping program, understanding the population structure and assessing the kinship relatedness is essential before making correlation between superior alleles and traits. The present study was aimed at evaluating the genetic variation and population structure in a collection of 192 rice germplasm lines including local landraces, improved varieties and exotic lines from diverse origin.

**Results:**

A set of 192 diverse rice germplasm lines were genotyped using 61 genome wide SSR markers to assess the molecular genetic diversity and genetic relatedness. Genotyping of 192 rice lines using 61 SSRs produced a total of 205 alleles with the PIC value of 0.756. Population structure analysis using model based and distance based approaches revealed that the germplasm lines were grouped into two distinct subgroups. AMOVA analysis has explained that 14 % of variation was due to difference between with the remaining 86 % variation may be attributed by difference within groups.

**Conclusions:**

Based on these above analysis viz., population structure and genetic relatedness, a core collection of 150 rice germplasm lines were assembled as an association mapping panel for establishing marker trait associations.

**Electronic supplementary material:**

The online version of this article (doi:10.1186/s12284-015-0062-5) contains supplementary material, which is available to authorized users.

## Background

Rice, being the staple food crop for more than 50 % of the world population is cultivated in 163 million hectares with the production of 491 million tonnes. About 90 % of the world’s rice is produced in Asia and India contributes 20 % of the world’s production. This record level production and productivity is due to the availability and exploitation of rich genetic diversity existing in rice germplasm of India. For precise genetic manipulation of complex quantitative traits like, yield, tolerance against biotic/abiotic stresses, quality etc., understanding the genetic/molecular basis of target traits needs to be investigated thoroughly.

The genetic basis of important agronomic traits has been unraveled through Quantitative Trait Loci (QTL) mapping either through linkage mapping (bi-parental mapping populations) or through LD mapping (natural populations). Although traditional linkage based QTL-mapping has become an important tool in gene tagging of crops, it has few limitations viz., 1) classical linkage mapping involves very high cost; 2) it has low resolution as it can resolve only a few alleles and 3) it has limitations towards fine mapping of QTLs as it needs BC-NILs. These limitations can be overcome by the LD based approach of “Association Mapping” using the natural populations. Association mapping serves as a tool to mine the elite genes by structuring the natural variation present in a germplasm. It was successfully exploited in various crops such as rice, maize, barley, durum wheat, spring wheat, sorghum, sugarcane, sugarbeet, soybean, grape, forest tree species and forage grasses (Abdurakhmonov and Abdukarimov 2008).

Before performing an association analysis in a population, it is essential to determine the population structure which can reduce type I and II errors in association mapping due to unequal allele frequency distribution between subgroups that causes spurious association between molecular markers and trait of interest (Pritchard et al. 2000). Similar attempts were recently undertaken to define population structure in rice using different germplasm lines and by developing core collection from national collections and international collections (Ebana et al. 2008; Jin et al. 2010; Zhang et al. 2011; Agrama et al. 2010 and Liakat Ali et al. 2011). Simple Sequence repeat (SSR) markers have been commonly used in genetic diversity studies in rice because of high level of polymorphism which helps to establish the relationship among the individuals even with less number of markers (McCouch et al. 1997). For similar studies, SSR markers were used alone by Jin et al. (2010); Hesham et al. (2008); Sow et al. (2014); Das et al. (2013) and Choudhury et al. (2013) or along with SNP markers by Courtois et al. (2012) and Zhao et al. (2011). The objectives of this present study were to evaluate the genetic variation and to examine the population structure of 192 rice germplasm accessions that comprises of local landraces, improved varieties and exotic lines from diverse origin.

## Results

### Genetic Diversity

All the 192 rice germplasm lines were genotyped using 61 SSR (microsatellite) markers which produced a total of 205 alleles (Additional file 1: Figure S1). Among these 205 alleles, 5 % were considered as rare (showed an allele frequency of < 5 %). The number of alleles per loci varied from 2 to 7 with an average of 3 alleles per locus. The highest number of alleles were detected for the loci RM316 (7) and the lowest was detected for a group of markers viz., RM171, RM284, RM455, RM514, RM277, RM 5795, HvSSR0247, RM 559, RM416 and RM1227. PIC value represents the relative informativeness of each marker and in the present study, the average PIC value was found to be 0.468. The highest genetic diversity is explained by the landraces included in this study with the mean PIC value of 0.416. PIC values ranged between 0.146 for RM17616 to 0.756 for RM316. Heterozygosity was found to be very low which may be due to autogamous nature of rice. Expected heterozygosity or Gene diversity (H_e_) computed according to Nei (1973) varied from 0.16 (RM17616) to 0.75 (RM287) with the average of 0.52 (Table 1).

**Table 1:** Details of SSR loci used for genotyping in the 192 rice accessions and their genetic diversity parameters

S. no	Marker	Chromosome no.	SSR MOTIF	Min molecular weight	Maximum molecular weight	Number of alleles	Gene diversity	Heterozygosity	PIC value
1	RM237	1	(CT)18	110	143	4	0.61	0.89	0.545
2	RM1	1	(GA)26	70	105	3	0.63	0.12	0.552
3	RM5	1	(GA)14	105	115	3	0.64	0.6	0.557
4	RM312	1	(ATTT)4(GT)9	95	105	3	0.3	0.03	0.281
5	RM283	1	(GA)18	149	155	3	0.42	0.02	0.377
6	RM452	2	(GTC)9	195	245	3	0.54	0.83	0.448
7	HvSSR0247	2		395	400	2	0.5	0.18	0.373
8	RM555	2	(AG)11	135	145	3	0.59	0.04	0.517
9	RM211	2	(TC)3A(TC)18	140	160	3	0.52	0.08	0.463
10	RM324	2	(CAT)21	135	180	5	0.74	0.06	0.695
11	RM514	3	(AC)12	245	252	2	0.19	0	0.171
12	RM55	3	(GA)17	220	225	3	0.44	0.07	0.4
13	RM231	3	(CT)16	170	200	3	0.59	0.12	0.511
14	RM416	3	(GA)9	110	115	2	0.42	0.01	0.335
15	RM442	3	(AAG)10	260	275	3	0.5	0.03	0.448
16	RM 16643	4	(GGGA)5	165	200	5	0.73	0.05	0.685
17	RM 559	4	(AACA)6	160	165	2	0.39	0.01	0.311
18	RM17377	4	(AG)25	140	175	4	0.67	0.04	0.625
19	RM7585	4	(TCTT)6	140	160	4	0.46	0.02	0.422
20	RM17616	4	(TC)14	165	180	3	0.16	0	0.146
21	RM413	5	(AG)11	75	100	4	0.59	0.25	0.548
22	RM178	5	(GA)5(AG)8	110	115	3	0.39	0.04	0.35
23	RM 161	5	(AG)20	160	180	3	0.29	0.04	0.258
24	RM7293	5	(ATGT)6	140	150	3	0.64	0.1	0.558
25	RM1024	5	(AC)13	125	140	3	0.32	0.02	0.298
26	RM 162	6	(AC)20	220	240	3	0.37	0.03	0.34
27	RM7434	6	(GTAT)10	120	145	5	0.66	0.19	0.614
28	RM19620	6	(GTG)7	160	177	3	0.21	0.03	0.204
29	RM5963	6	(CAG)9	160	175	3	0.48	0.15	0.38
30	RM11	7	(GA)17	120	150	4	0.71	0.72	0.661
31	RM118	7	(GA)8	155	185	4	0.62	0.77	0.543
32	RM125	7	(GCT)8	105	130	4	0.61	0.89	0.544
33	RM455	7	(TTCT)5	130	135	2	0.24	0.02	0.208
34	HvSSR0740	7		340	400	4	0.7	0.21	0.65
35	RM44	8	(GA)16	95	107	4	0.62	0.77	0.559
36	RM433	8	(AG)13	235	270	3	0.55	0.81	0.446
37	RM447	8	(CTT)8	105	120	4	0.64	0.16	0.572
38	RM284	8	(GA)8	140	145	2	0.21	0.02	0.189
39	RM408	8	(CT)13	120	125	3	0.52	0.01	0.465
40	RM25	8	(GA)18	120	140	4	0.73	0.37	0.679
41	RM256	8	(CT)21	125	140	4	0.73	0	0.681
42	RM105	9	(CCT)6	100	140	3	0.41	0.48	0.37
43	RM107	9	(GA)7	280	300	3	0.48	0	0.425
44	RM 215	9	(CT)16	140	150	3	0.6	0.01	0.528
45	RM 316	9	(GT)8-(TG)9(TTTG)4(TG)4	160	235	7	0.79	0.75	0.756
46	RM205	9	(CT)25	110	140	4	0.72	0	0.665
47	RM171	10	(GATG)5	320	330	2	0.24	0.02	0.211
48	RM271	10	(GA)15	90	99	3	0.66	0.19	0.588
49	RM590	10	(TCT)10	120	140	4	0.57	0.04	0.516
50	RM474	10	(AT)13	240	280	3	0.61	0	0.537
51	RM222	10	(CT)18	200	220	3	0.63	0.02	0.557
52	RM144	11	(ATT)11	160	240	5	0.69	0.18	0.644
53	RM287	11	(GA)21	95	110	5	0.75	0.2	0.706
54	RM 536	11	(CT)16	240	270	5	0.74	0.06	0.701
55	RM224	11	(AAG)8(AG)13	120	155	5	0.65	0.07	0.617
56	RM206	11	(CT)21	130	145	4	0.34	0	0.319
57	RM277	12	(GA)11	115	120	2	0.45	0.08	0.35
58	RM 5795	12	(AGC)8	140	145	2	0.5	0.03	0.374
59	RM1227	12	(AG)15	160	180	2	0.31	0.02	0.262
60	RM20A	12	(ATT)14	220	240	3	0.54	0	0.476
61	RM2197	12	(AT)23	135	140	2	0.44	0	0.341
	Average					3	0.52	0.18	0.468

### STRUCTURE Analysis

**Fig. 1:**
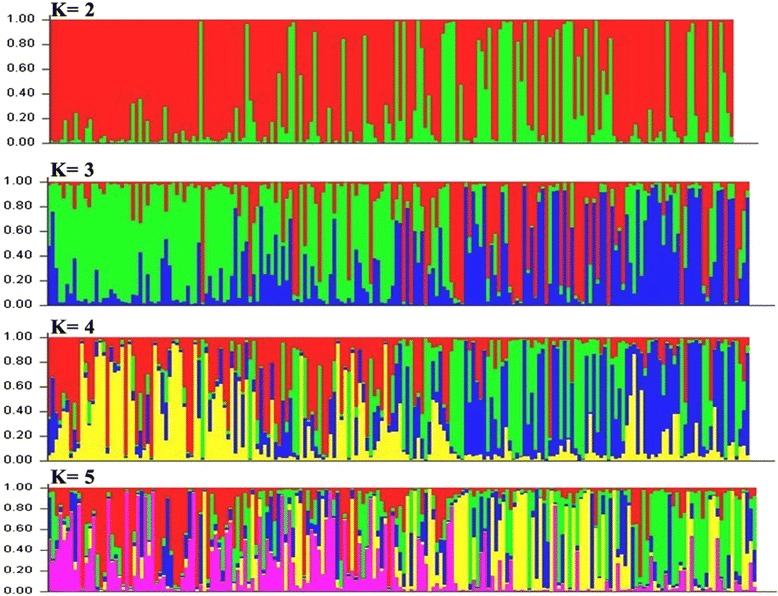
Pattern of variation of 192 accessions based on 61 SSR markers. The K values are based on the run with highest likelihood. Bar length represent the membership probability of accessions belonging to different subgroups

**Fig. 2:**
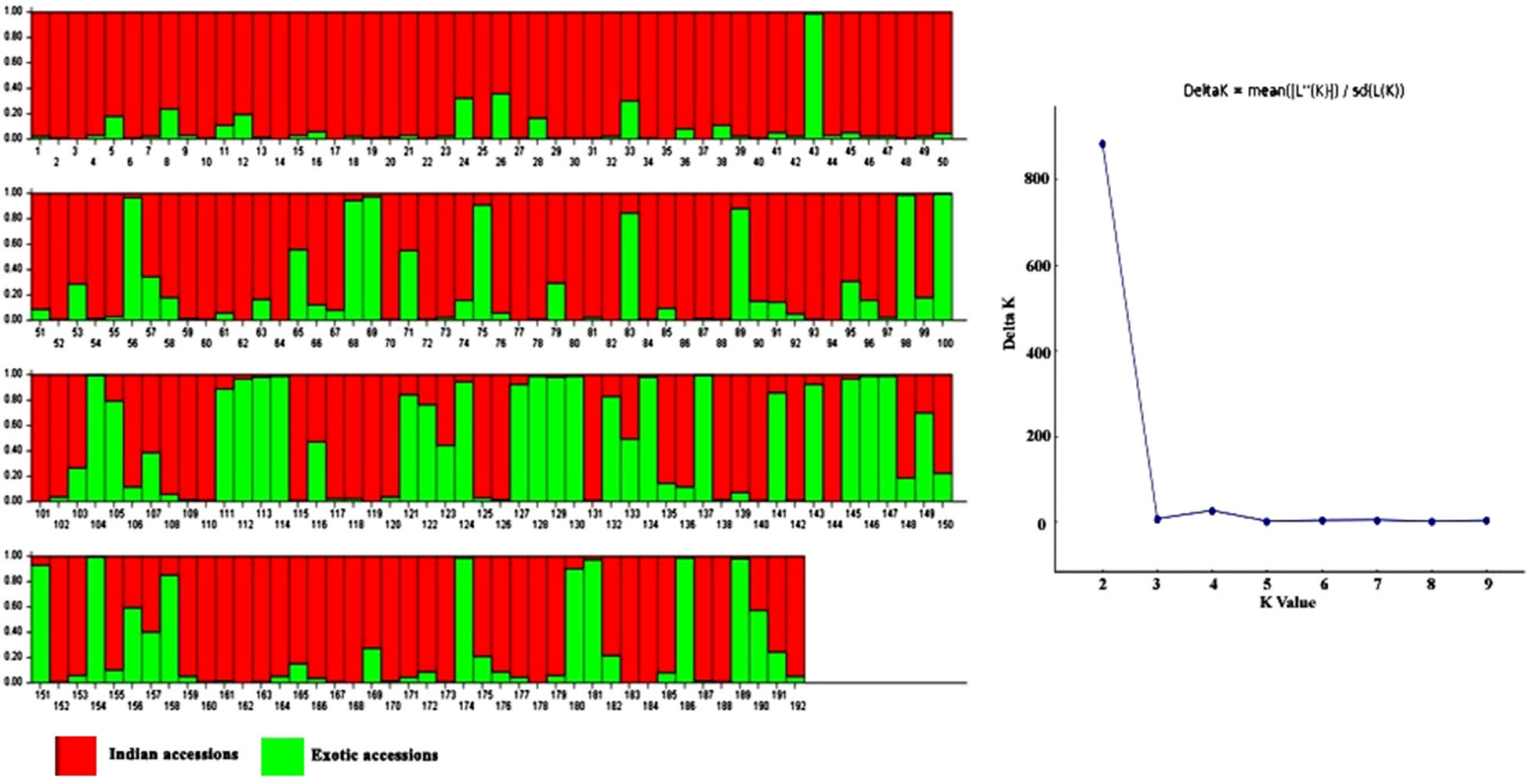
Population structure of 192 accessions based on 61 SSR markers (*K* = 2) and Graph of estimated membership fraction for *K* = 2. The maximum of adhoc measure ΔK determined by structure harvester was found to be *K* = 2, which indicated that the entire population can be grouped into two subgroups (SG1 and SG2)

**Fig. 3:**
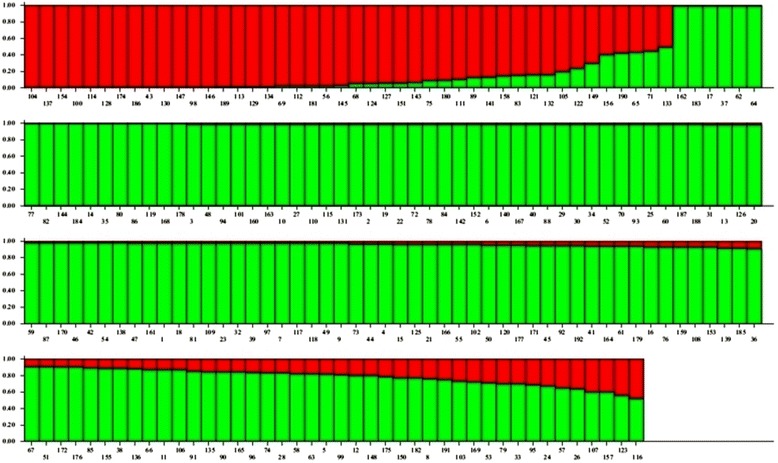
Population structure of 192 accessions arranged based on inferred ancestry. Based on the membership fractions, the accessions with the probability of ≥ 80 % were assigned to corresponding subgroups with others categorized as admixture

Population structure of the 192 germplasm lines was analysed by Bayesian based approach. The estimated membership fractions of 192 accessions for different values of k ranged between 2 and 5 (Fig. 1). The log likelihood revealed by structure showed the optimum value as 2 (*K* = 2). Similarly the maximum of adhoc measure ΔK was found to be *K* = 2 (Fig. 2), which indicated that the entire population can be grouped into two subgroups (SG1 and SG2). Based on the membership fractions, the accessions with the probability of ≥ 80 % were assigned to corresponding subgroups with others categorized as admixture (Fig. 3).

**Fig. 4:**
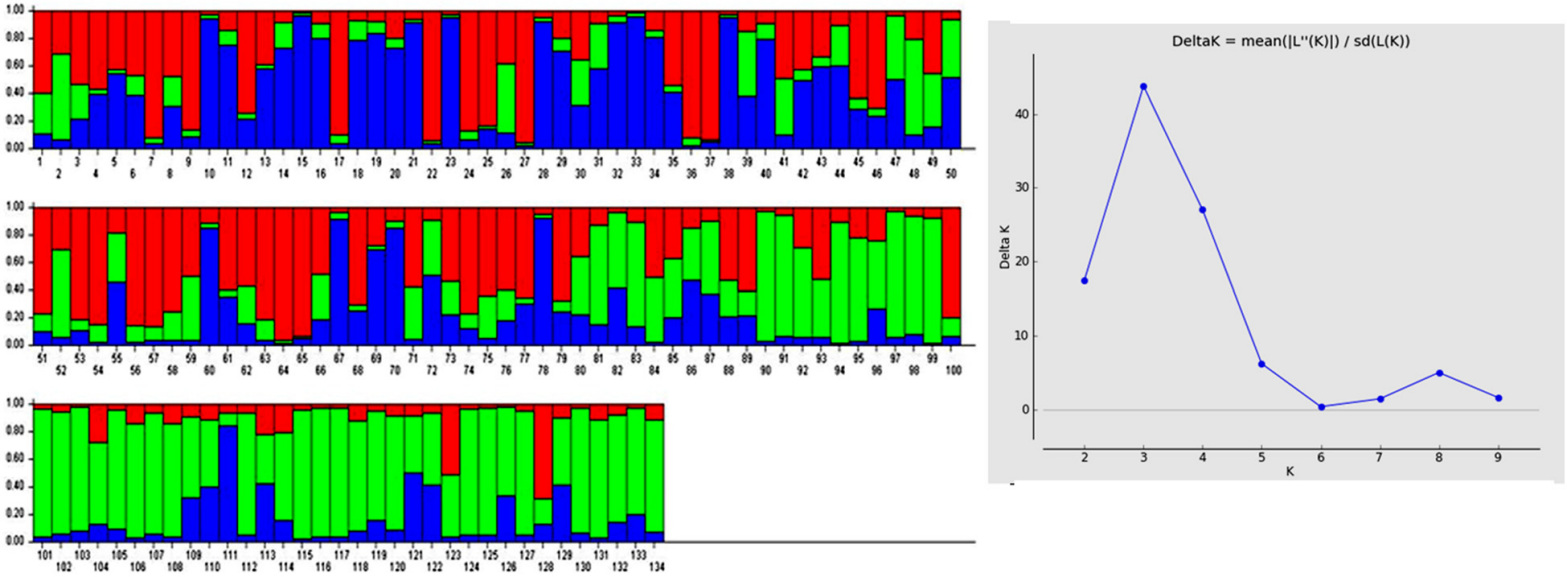
Population structure of 134 accessions in sub group-1 and membership probability of assigning genotypes of sub group-1 (*K* = 3)

SG1 consisted of 134 accessions with most of the landraces and varieties of Indian origin and SG2 consisted of 38 accessions which composed of non Indian accessions. Twenty accessions were retained to be admixture. The subgroup SG1 was dominated by *indica* subtype whereas the subgroup SG2 consisted mostly of *japonica* group. When the number of subgroups increased from two to five, the accessions in both the subgroups were classified into sub-sub groups (Table 2). As SG1 consisted of 134 accessions mostly of Indian origin, an independent STRUCTURE analysis was performed for this subgroup. ΔK showed its maximum value for K =3 which indicated that SG1 could be further classified into three sub-sub groups (Fig. 4). The differentiation in origin and seasonal differentiation of rice varieties contributed for this clustering.

**Table 2:** Population structure group of accessions based on Inferred ancestry values

G. no.	Genotypes	Inferred ancestry		Structure group	Subtype
		Q1	Q2		
RG1	Mapillai samba	0.977	0.023	SG1	*Indica*
RG2	CK 275	0.991	0.009	SG1	*Indica*
RG3	Senkar	0.992	0.008	SG1	*Indica*
RG4	Murugankar	0.964	0.036	SG1	*Indica*
RG5	CHIR 6	0.811	0.189	SG1	*Indica*
RG6	CHIR 5	0.989	0.011	SG1	*Indica*
RG7	Kudai vazhai	0.975	0.025	SG1	*Indica*
RG8	CHIR 8	0.759	0.241	SG1	*Indica*
RG9	Kuruvai kalanjiyam	0.971	0.029	SG1	*Indica*
RG10	Nava konmani	0.99	0.01	SG1	*Indica*
RG11	CHIR 10	0.869	0.131	SG1	*Indica*
RG12	Vellai chithiraikar	0.802	0.198	SG1	*Indica*
RG13	CHIR 2	0.983	0.017	SG1	*Indica*
RG14	Jothi	0.992	0.008	SG1	*indica*
RG15	Palkachaka	0.962	0.038	SG1	*indica*
RG16	Thooyala	0.934	0.066	SG1	*indica*
RG17	Chivapu chithiraikar	0.994	0.006	SG1	*indica*
RG18	CHIR 11	0.976	0.024	SG1	*indica*
RG19	Koolavalai	0.99	0.01	SG1	*indica*
RG20	Kalvalai	0.982	0.018	SG1	*indica*
RG21	Mohini samba	0.963	0.037	SG1	*indica*
RG22	IR 36	0.989	0.011	SG1	*indica*
RG23	Koombalai	0.975	0.025	SG1	*indica*
RG24	Tadukan	0.674	0.326	AD	*indica*
RG25	Sorna kuruvai	0.986	0.014	SG1	*indica*
RG26	Rascadam	0.637	0.363	AD	*indica*
RG27	Muzhi karuppan	0.991	0.009	SG1	*indica*
RG28	Kaatukuthalam	0.828	0.172	SG1	*indica*
RG29	Vellaikattai	0.987	0.013	SG1	*indica*
RG30	Poongar	0.987	0.013	SG1	*indica*
RG31	Chinthamani	0.985	0.015	SG1	*indica*
RG32	Thogai samba	0.975	0.025	SG1	*indica*
RG33	Malayalathan samba	0.701	0.299	AD	*indica*
RG34	RPHP 125	0.986	0.014	SG1	*indica*
RG35	CK 143	0.993	0.007	SG1	*indica*
RG36	Kattikar	0.913	0.087	SG1	*indica*
RG37	Shenmolagai	0.994	0.006	SG1	*indica*
RG38	Velli samba	0.887	0.113	SG1	*indica*
RG39	Kaatu ponni	0.975	0.025	SG1	*indica*
RG40	kakarathan	0.989	0.011	SG1	*indica*
RG41	Godavari samba	0.941	0.059	SG1	*indica*
RG42	Earapalli samba	0.978	0.022	SG1	*indica*
RG43	RPHP 129	0.01	0.99	SG2	*indica*
RG44	Mangam samba	0.968	0.032	SG1	*indica*
RG45	RPHP 105	0.943	0.057	SG1	*indica*
RG46	IG 4(EC 729639- 121695)	0.977	0.023	SG1	*indica*
RG47	Machakantha	0.976	0.024	SG1	*indica*
RG48	Kalarkar	0.992	0.008	SG1	*indica*
RG49	Valanchennai	0.972	0.028	SG1	*indica*
RG50	Sornavari	0.957	0.043	SG1	*indica*
RG51	RPHP 134	0.909	0.091	SG1	*indica*
RG52	ARB 58	0.987	0.013	SG1	*indica*
RG53	IR 68144-2B-2-2-3-1-127	0.708	0.292	AD	*indica*
RG54	PTB 19	0.981	0.019	SG1	*indica*
RG55	IG 67(EC 729050- 120988)	0.957	0.043	SG1	*indica*
RG56	RPHP 59	0.031	0.969	SG2	*Aromatic*
RG57	RPHP 103	0.656	0.344	AD	*Aromatic*
RG58	Kodaikuluthan	0.828	0.172	SG1	*indica*
RG59	RPHP 68	0.981	0.019	SG1	*indica*
RG60	Rama kuruvaikar	0.985	0.015	SG1	*indica*
RG61	Kallundai	0.939	0.061	SG1	*indica*
RG62	Purple puttu	0.994	0.006	SG1	*indica*
RG63	IG 71(EC 728651- 117588)	0.823	0.177	SG1	*aus*
RG64	Ottadaiyan	0.994	0.006	SG1	*indica*
RG65	IG 56(EC 728700- 117658	0.435	0.565	AD	*Aromatic*
RG66	Jeevan samba	0.876	0.124	SG1	*indica*
RG67	RPHP 106	0.915	0.085	SG1	*indica*
RG68	IG 63(EC 728711- 117674)	0.049	0.951	SG2	*Tropical Japonica*
RG69	RPHP 48	0.025	0.975	SG2	*Aromatic*
RG70	Karthi samba	0.987	0.013	SG1	*indica*
RG71	IG 27(IC 0590934- 121255)	0.444	0.556	AD	*indica*
RG72	Aarkadu kichili	0.99	0.01	SG1	*indica*
RG73	Kunthali	0.969	0.031	SG1	*indica*
RG74	ARB 65	0.83	0.17	SG1	*indica*
RG75	IG 21(EC 729334- 121355)	0.091	0.909	SG2	*japonica*
RG76	Matta kuruvai	0.934	0.066	SG1	*indica*
RG77	Karuthakar	0.994	0.006	SG1	*indica*
RG78	RPHP 165	0.99	0.01	SG1	*indica*
RG79	Manavari	0.704	0.296	AD	*indica*
RG80	IG 66(EC 729047- 120985)	0.992	0.008	SG1	*indica*
RG81	CB-07-701-252	0.977	0.023	SG1	*indica*
RG82	Thooyamalli	0.994	0.006	SG1	*indica*
RG83	RPHP 93	0.153	0.847	SG2	*indica*
RG84	Velsamba	0.99	0.01	SG1	*indica*
RG85	RPHP 104	0.898	0.102	SG1	*indica*
RG86	RPHP 102	0.993	0.007	SG1	*indica*
RG87	IG 40(EC 728740- 117705)	0.98	0.02	SG1	*indica*
RG88	Saranga	0.988	0.012	SG1	*indica*
RG89	IR 83294-66-2-2-3-2	0.125	0.875	SG2	*japonica*
RG90	IG 61(EC 728731- 117696)	0.843	0.157	SG1	*indica*
RG91	IG 23(EC 729391- 121419)	0.852	0.148	SG1	*Aus*
RG92	IG 49(EC 729102- 121052)	0.945	0.055	SG1	*indica*
RG93	uppumolagai	0.987	0.013	SG1	*indica*
RG94	Karthigai samba	0.993	0.007	SG1	*indica*
RG95	Jeeraga samba	0.685	0.315	SG1	*indica*
RG96	RP-BIO-226	0.833	0.167	SG1	*indica*
RG97	Varigarudan samba	0.975	0.025	SG1	*indica*
RG98	IG 5(EC 729642- 121698)	0.012	0.988	SG2	*japonica*
RG99	IG 31(EC 728844- 117829)	0.813	0.187	SG1	*indica*
RG100	IG 7(EC 729598- 121648)	0.008	0.992	SG2	*japonica*
RG101	RPHP 52	0.991	0.009	SG1	*indica*
RG102	Varakkal	0.958	0.042	SG1	*indica*
RG103	Mattaikar	0.732	0.268	AD	*indica*
RG104	IG 53(EC 728752- 117719)	0.005	0.995	SG2	*Temperate japonica*
RG105	IG 6(EC 729592- 121642)	0.204	0.796	SG2	*Temperate japonica*
RG106	Katta samba	0.872	0.128	SG1	*indica*
RG107	RH2-SM-1-2-1	0.606	0.394	AD	*indica*
RG108	Red sirumani	0.93	0.07	SG1	*indica*
RG109	Vadivel	0.977	0.023	SG1	*indica*
RG110	Norungan	0.991	0.009	SG1	*indica*
RG111	IG 20(EC 729293- 121310)	0.113	0.887	SG2	*indica*
RG112	IG 35(EC 728858- 117843)	0.027	0.973	SG2	*japonica*
RG113	IG 45(EC 728768- 117736)	0.017	0.983	SG2	*japonica*
RG114	RPHP 159	0.008	0.992	SG2	aromatic rice
RG115	IG 43(EC 728788- 117759)	0.992	0.008	SG1	*indica*
RG116	RPHP 27	0.52	0.48	AD	*Tropical Japonica*
RG117	IG 65(EC 729024- 120958)	0.974	0.026	SG1	*indica*
RG118	Ponmani samba	0.973	0.027	SG1	*indica*
RG119	Ganthasala	0.993	0.007	SG1	*indica*
RG120	Thattan samba	0.949	0.051	SG1	*indica*
RG121	IG 74(EC 728622- 117517)	0.16	0.84	SG2	*japonica*
RG122	Kaliyan samba	0.245	0.755	AD	*indica*
RG123	IG 2(EC 729808-121874)	0.56	0.44	AD	*japonica*
RG124	IG 29(EC 728925- 117920)	0.059	0.941	SG2	*Tropical Japonica*
RG125	RPHP 55	0.963	0.037	SG1	*indica*
RG126	Kallimadayan	0.984	0.016	SG1	*indica*
RG127	IG 10(EC 729686- 121743)	0.066	0.934	SG2	*aromatic*
RG128	IG 75(EC 728587- 117420)	0.008	0.992	SG2	*japonica*
RG129	IG 38(EC 728742 - 117707)	0.02	0.98	SG2	*Tropical japonica*
RG130	IG 39(EC 728779- 117750)	0.012	0.988	SG2	*indica*
RG131	RPHP 90	0.991	0.009	SG1	*indica*
RG132	IG 33(EC 728938- 117935)	0.162	0.838	SG2	*Tropical Japonica*
RG133	IG 42(EC 728798- 117774)	0.495	0.505	AD	*indica*
RG134	IG 9(EC 729682- 121739)	0.019	0.981	SG2	*indica*
RG135	RPHP 161	0.849	0.151	SG1	*indica*
RG136	IG 8(EC 729601- 121651)	0.883	0.117	SG1	*indica*
RG137	IG 37(EC 728715- 117678)	0.005	0.995	SG2	*Tropical Japonica*
RG138	Sigappu kuruvikar	0.979	0.021	SG1	*indica*
RG139	RPHP 138	0.917	0.083	SG1	*indica*
RG140	Raja mannar	0.989	0.011	SG1	*indica*
RG141	IG 44(EC 728762- 117729)	0.134	0.866	SG2	*indica*
RG142	Sasyasree	0.989	0.011	SG1	*indica*
RG143	IG 46(IC 471826- 117647)	0.073	0.927	SG2	*indica*
RG144	Chetty samba	0.993	0.007	SG1	*indica*
RG145	IG 60(EC 728730- 117695)	0.033	0.967	SG2	*indica*
RG146	IR 75862-206	0.013	0.987	SG2	*Tropical Japonica*
RG147	IG 58(EC 728725- 117689)	0.011	0.989	SG2	*japonica*
RG148	Chinna aduku nel	0.798	0.202	SG1	*indica*
RG149	RH2-SM-2-23	0.296	0.704	AD	*indica*
RG150	IG 14(IC 517381- 121422)	0.775	0.225	AD	*indica*
RG151	IG 32(EC 728838- 117823)	0.065	0.935	SG2	*japonica*
RG152	RPHP 47	0.989	0.011	SG1	*indica*
RG153	Sembilipiriyan	0.933	0.067	SG1	*indica*
RG154	IG 48(EC 729203- 121195)	0.006	0.994	SG2	*indica*
RG155	Sona mahsuri	0.889	0.111	SG1	*indica*
RG156	IG 12(EC 729626- 121681)	0.405	0.595	AD	*indica*
RG157	Karungan	0.602	0.398	AD	*indica*
RG158	IG 13(EC 729640- 121696)	0.143	0.857	SG2	*indica*
RG159	Sembala	0.934	0.066	SG1	*indica*
RG160	IG 72(EC 728650- 117587)	0.992	0.008	SG1	*indica*
RG161	Panamarasamba	0.978	0.022	SG1	*indica*
RG162	IR 64	0.995	0.005	SG1	*indica*
RG163	Mikuruvai	0.992	0.008	SG1	*indica*
RG164	Thillainayagam	0.939	0.061	SG1	*indica*
RG165	ARB 64	0.843	0.157	SG1	*indica*
RG166	RPHP 140	0.959	0.041	SG1	*indica*
RG167	IG 70(EC 729045- 120983)	0.989	0.011	SG1	*indica*
RG168	Haladichudi	0.993	0.007	SG1	*indica*
RG169	IG 24(EC 728751- 117718)	0.725	0.275	AD	*Aus*
RG170	RPHP 42	0.981	0.019	SG1	*indica*
RG171	RPHP 44	0.951	0.049	SG1	*indica*
RG172	IG 25(EC 729728- 121785)	0.903	0.097	SG1	*Tropical Japonica*
RG173	IG 73(EC 728627- 117527)	0.991	0.009	SG1	*indica*
RG174	IG 51(EC 728772- 117742)	0.008	0.992	SG2	*Tropical Japonica*
RG175	Vellai kudaivazhai	0.786	0.214	SG1	*indica*
RG176	Kodai	0.906	0.094	SG1	*indica*
RG177	Kallundaikar	0.951	0.049	SG1	*indica*
RG178	IG 17(EC 728900- 117889)	0.993	0.007	SG1	*indica*
RG179	Avasara samba	0.939	0.061	SG1	*indica*
RG180	IG 59(EC 728729- 117694)	0.093	0.907	SG2	*Tropical Japonica*
RG181	IG 52(EC 728756- 117723)	0.026	0.974	SG2	*Tropical Japonica*
RG182	ARB 59	0.779	0.221	SG1	*indica*
RG183	RPHP 163	0.995	0.005	SG1	*indica*
RG184	IG 18(EC 728892- 117880)	0.994	0.006	SG1	*indica*
RG185	RPHP 36	0.915	0.085	SG1	*indica*
RG186	IG 28(EC 728920- 117914)	0.009	0.991	SG2	*Tropical Japonica*
RG187	Vadakathi samba	0.986	0.014	SG1	*indica*
RG188	RPHP 80	0.986	0.014	SG1	*indica*
RG189	IG 41(EC 728800- 117776)	0.016	0.984	SG2	*Tropical japonica*
RG190	IG 26(IC 0590943- 121899)	0.422	0.578	SG2	*aromatic*
RG191	IG 15(EC 728910- 117901)	0.755	0.245	AD	*indica*
RG192	Nootri pathu	0.943	0.057	SG1	*indica*

**Fig. 5:**
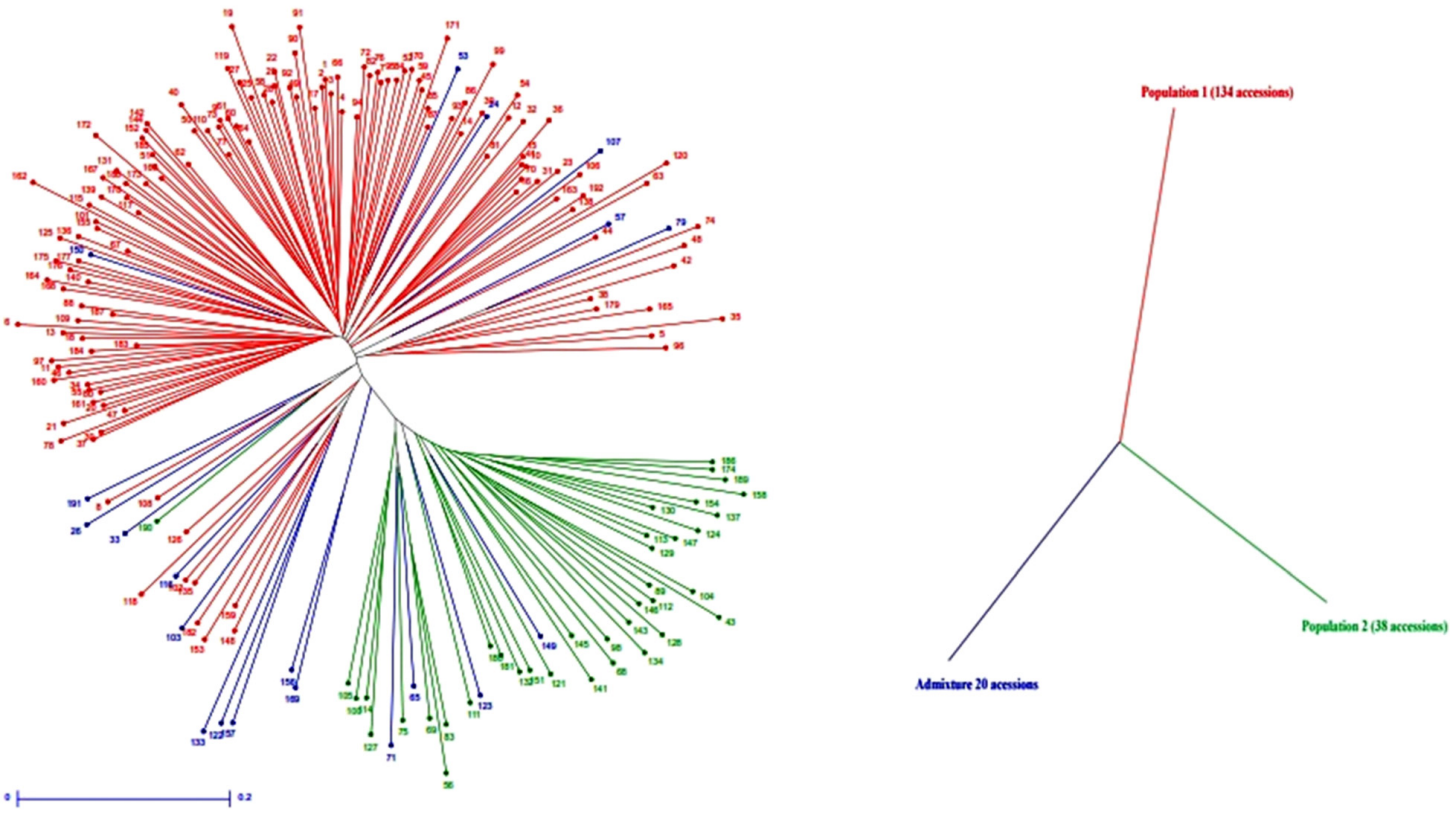
Unrooted neighbour joining tree of 192 rice varieties. The landraces and varieties used in the study has clustered in the upper branches of the tree whereas the exotic accessions has positioned in the lower branches of the tree

**Fig. 6:**
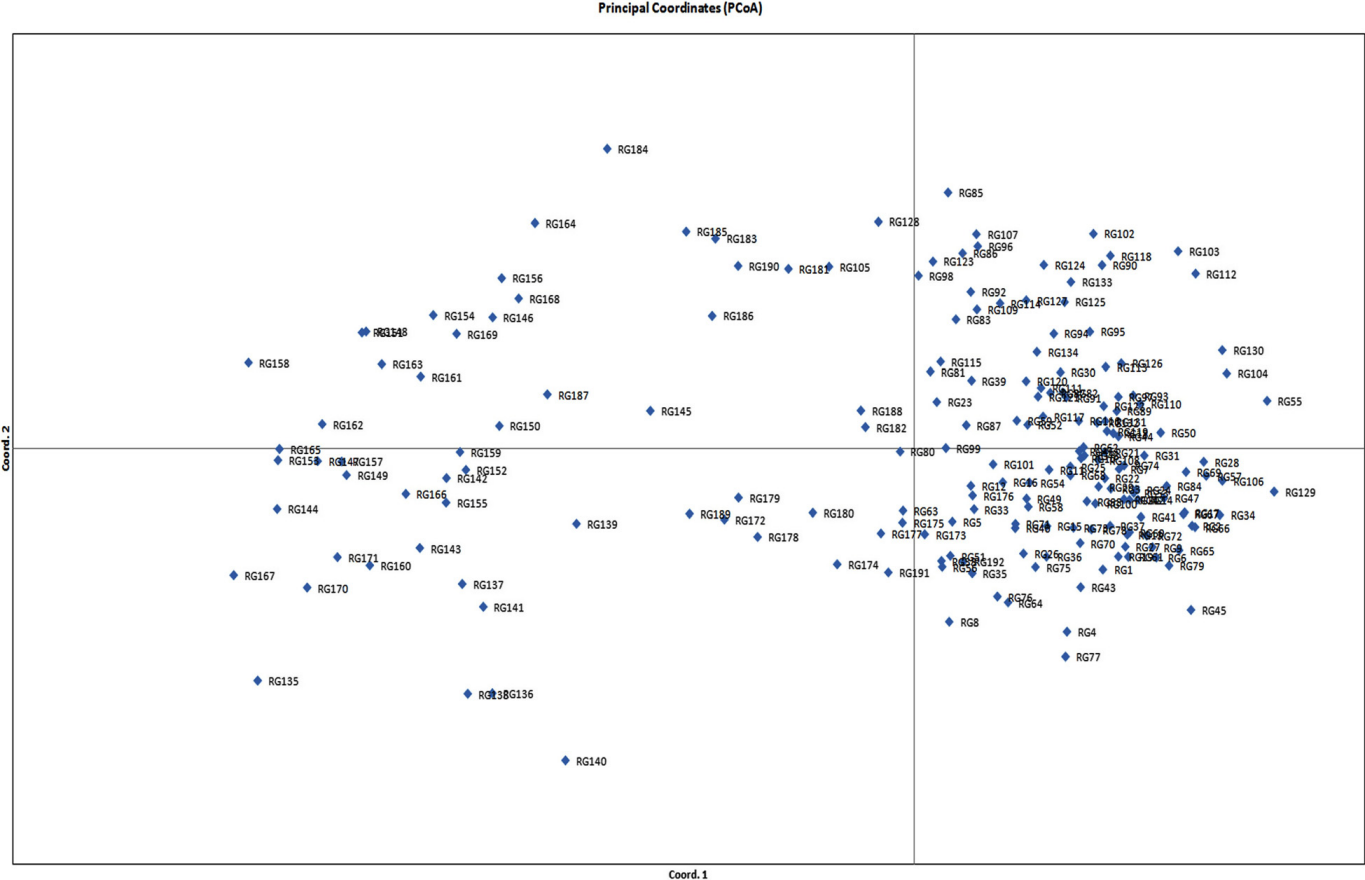
Principal Coordinates of 192 accessions based on 61 SSR loci. Coord 1 and Coord 2 represent first and second coordinates, respectively. The two PCA axes accounted for 12.6 and 4.9 % of the genetic variation among populations

Clustering analysis based on Unweighted Pair Group Method with Arithmetic Mean (UPGMA) method using DARwin separated the accessions into two main groups which showed similar results as STRUCTURE analysis. The group I in UPGMA tree consists of both indigenous and agronomically improved varieties whereas the other group consists of exotic accessions. In UPGMA tree, the accessions within group 1 and 2 clustered into smaller sub groups based on their origin and types. Most of the landraces and varieties have been clustered in upper branches of the tree whereas the exotic accessions have been clustered in lower branches of the tree (Fig 5). Hence the clustering analysis by two classification methods revealed high level of similarity in clustering the genotypes. PCoA was used to characterize the subgroups of the germplasm set. A two- dimensional scatter plot involving all 192 accessions has shown that the first two PCA axes accounted for 12.6 and 4.9 % of the genetic variation among populations (Fig 6).

### Genetic Variance Analysis

The hierarchial distribution of molecular variance by AMOVA and pair-wise analysis revealed highly significant genetic differentiation among the groups. It revealed that 14 % of the total variation was between the groups, while 86 % was among individuals within groups (Tables 3 and 4). Calculation of Wright’s F statistic at all SSR loci revealed that F_IS_ was 0.50 and F_IT_ was 0.56. Determination of F_ST_ for the polymorphic loci across all accessions has shown F_ST_ as 0.14 which implies high genetic variation (Table 4). The pairwise F_ST_ estimate among sub-groups has indicated that the two groups are significantly different from each other (Table 3).

**Table 3:** AMOVA between groups and Pair wise comparison using Fst values (GenAlEx)

Source	df	SS	MS	Est. var.	Percent
Among the population	2	971.922	485.961	9.631	14 %
Within Pops	189	10961.256	57.996	57.996	86 %
Total	191	11933.177		67.627	100 %
Pairwise population Fst values					
	SG2		AD		
SG1	0.128		0.040		
SG2			0.061

**Table 4:** AMOVA between groups and accessions and Fixation indices (Arlequin software)

Source of variation	d.f.	Sum of squares	Variance components	Percentage of variation
Among Populations	2	200.013	1.01840 Va	13.82
Among individuals within Populations	189	1794.771	3.14391 Vb	42.65
Within Individuals	192	616	3.20833 Vc	43.53
	383	2610.784	7.37064	
Fixation Indices				
FIS	0.49493			
FST	0.13817			
FIT	0.56471			

## Discussion

Genetic diversity is the key determinant of germplasm utilization in crop improvement. Population with high level of genetic variation is the valuable resource for broadening the genetic base in any breeding program. The panel of 192 accessions in this study with landraces, varieties as well as breeding lines has different salient agronomic traits. Few landraces included in this study *i.e.,* Mappillai samba (Krishnanunni et al. 2015), Jyothi, Njavara (Deepa et al. 2008), Kavuni (Valarmathi et al. 2015) derived breeding line has therapeutic properties. Many lines included in this study are drought tolerant (Nootripathu, Norungan, Vellaikudaivazhai, kallundaikar, kodai, kalinga 3, Kinandang patong, azucena, mattaikar, IR65907-116-1, karuthakar, mattakuruvai, manavari, kallundai, kodaikulathan, kattikar, poongar, thogai samba, vellaikattai, kattukuthalam, kalvalai, chivapu chithiraikar, vellai chithiraikar, kudaivazhai and murugankar). Few lines have significant level of micronutrients in it (Nachimuthu et al. 2014). This panel has its importance because of its major component as traditional landraces with valuable agronomic traits that are cultivated in the small pockets of Tamil Nadu, India.

Molecular markers help us to understand the level of genetic diversity that exists among traditional races, varieties and exotic accessions which can be exploited in rice breeding programs. The genetic architecture of diverse germplasm lines can be precisely estimated by assessing the STRUCTURE of the population using molecular markers viz., SSRs or SNPs etc., (Horst and Wenzel 2007; Powell et al. 1996; Varshney et al. 2007). In this study, the genetic diversity among the accessions was evaluated by model based clustering and distance based clustering approach using the SSR genotypic data.

Regarding genetic divergence of the population consisting of local landraces, exotic cultivars and breeding lines, 61 polymorphic markers have detected a total of 205 alleles across 192 individuals. The number of alleles varied from 2 to 7 per locus and the average was 3 alleles per locus. Several previous reports have indicated the number of alleles per locus, polymorphic information content and gene diversity of 4.8–14.0, 0.63–0.70 and 6.2–6.8 respectively (Garris et al. 2005; Ram et al. 2007). In the current study, the average number of alleles (3 alleles/locus) is slightly lesser than the average number of alleles (3.88 alleles/ locus) reported by Zhang et al. (2011) in rice core collection with 150 rice varieties from south Asia and Brazil and Jin et al. (2010) who has reported the average alleles per locus as 3.9 in 416 rice accessions collected from China. Using three sets of germplasm lines (Thai (47), IRRI germplasm (53) amd other Oryza species (5)), Chakhonkaen et al. (2012) has reported 127 alleles for all loci, with a mean of 6.68 alleles per locus, and a mean Polymorphic Information Content (PIC) of 0.440 by screening with 19 InDel markers.

Chen et al. (2011) has reported the average gene diversity of 0.358 and polymorphic information content of 0.285 from 300 rice accessions from different rice growing areas of the world with 372 SNP markers. The gene diversity detected in this study (0.52) is comparable to overall gene diversity of rice core collection (0.544) from China, North Korea, Japan, Philippines, Brazil, Celebes, Java, Oceanina and Vietnam (Zhang et al. 2011) and it is higher than US accession panel with average gene diversity of 0.43 (Agrama and Eizenga 2008) and Chinese rice accession panel by Jin et al. (2010) with the average gene diversity of 0.47. The gene diversity reported in our study is lesser than gene diversity (0.68) reported by (Liakat Ali et al. 2011). Most of the diversity panel with global accessions has the gene diversity of 0.5 to 0.7 (Garris et al. 2005; Liakat Ali et al. 2011; Ni et al. 2002). These results on global accessions help to infer that this diversity panel of 192 germplasm lines represents a large proportion of the genetic diversity that exists in major rice growing Asian continent.

The PIC value was 0.468 which varied from 0.146 for RM17616 with only 2 two alleles to 0.756 for RM316 that allowed the amplification of 7 alleles. The PIC value was found to be 0.418 for SG1 which had the majority of *indica* accessions. The subgroup SG2 dominated by japonica accessions had the PIC value of 0.414. Hence, both the subgroups contribute in a major way for population diversity. As this population encompass different rice materials *i.e.,* landraces, varieties and breeding lines, the molecular diversity is contributed majorly by landraces. These values are similar to those found by Courtois et al. (2012) who reported the PIC value from 0.16 to 0.78 with the average of 0.49 in European rice germplasm collection and in Chinese rice collection of 416 accessions by Jin et al. (2010), who has given similar PIC value of 0.4214. It is also consistent with PIC value (0.48) attained by Zhang et al. (2011). In this study, significant amount of rare alleles was identified which indicates that these rare alleles contribute well to the overall genetic diversity of the population.

Model based approach by STRUCTURE is implemented frequently for studying population structure by various researchers (Agrama et al. 2007, Agrama and Eizenga 2008; Garris et al. 2005; Zhang et al. 2007, 2011; Jin et al. 2010; Liakat Ali et al. 2011, Chakhonkaen et al. 2012 Courtois et al. 2012, Das et al. 2013). Courtois et al. (2012) has successfully detected two subgroups in their study population and assigned rice varieties into two groups with few admixture lines. Jin et al. (2010) has identified seven sub populations among 416 rice accessions from China. Das et al. (2013) has grouped a collection of 91 accessions of rice landraces from eastern and north eastern India into four groups.

Assigning of genotypes to the subgroups based on ancestry threshold vary between different research groups. Zhao et al. (2010) and Courtois et al. (2012) used an ancestry threshold of 80 % to identify accessions belonging to a specific subpopulation. Liakat Ali et al. (2011) has steup the threshold as 60 % and identified 33 accessions as admixtures as the threshold of 80 % consider more genotypes as admixtures. In the current study, a stringent threshold of 80 % ancestry value leaves only 20 genotypes as admixtures.

Population structure analysis in different rice diversity panel has indicated the existence of two to eight sub population in rice (Zhang et al. 2007, Zhang et al. 2009, Zhang et al. 2011, Garris et al. 2005, Agrama et al. 2007, Liakat Ali et al. 2011, Chakhonkaen et al. 2012 and Das et al. 2013). In the current rice diversity panel of 192 accessions based on the criterion of maximum membership probabilities, 134 accessions were assigned to SG1 which is dominated by *indica* subtype with most of the landraces and varieties of Indian origin and SG2 consisted of 38 accessions which composed mostly of *japonica* accessions of exotic origin. Similar population structure of two subgroups was observed in previous research by Zhang et al. (2009) in a collection of 3024 rice landraces in China. Zhang et al. (2011) has reported two distinct subgroups in a rice core collection. Courtois et al. (2012) has successfully classified two subgroups as japonica and non japonica accessions in European core collection of rice. The results indicated that two subgroups are due to the different adaptation behavior of accessions to different ecological environment as *indica* and *japonica* accessions has independent evolution frame and the origin of Indian rice accessions from *indica* cultivars. Hence the major criterion for population structure in this panel is *indica – japonica* subtype. This study includes large number of traditional landraces and varieties from Indian Subcontinent and few exotic accessions randomly selected from IRRI worldwide collection. It clarifies the relationship between Indian germplasm and exotic accessions which indicates that germplasm lines varies based on its ecology and also shows higher level of genetic diversity exists within this population.

Further structure analysis of SG1 that consisted of 134 lines indicated that it can be further subdivided in to three sub sub-groups. The three sub sub-groups classification has the factor of ecosystem and seasonal variation as the major factors for population structure. This results is in accordance with the inference that indica group has higher genetic diversity than japonica accessions which was given by various researchers (Gao et al. 2005; Lu et al. 2005; Lapitan et al. 2007; Caicedo et al. 2007; Liakat Ali et al. 2011; Garris et al. 2005; Qi et al. 2006; Qi et al. 2009); as this subgroup has indica accessions. Liakat Ali et al. (2011) has substantiated this statement with the reason of the indica subpopulation occupying the largest rice growing region which has a varied environments, ecological conditions and soil type.

The result of model based analysis is in accordance with the clustering pattern of Neighbour joining tree and Principal Coordinate Analysis. The first two principal coordinates explained 12.6 and 4.8 % of the molecular variance. Similar pattern of molecular variance explanation was observed by Zhang et al. (2011) for two population subgroups.

Calculation of Wright’s F Statistic at all loci revealed the deviation from Hardy- Weinberg law for molecular variation within the population. The result of F_st_ indicates higher divergence existing between subgroups of the population. Higher F_IT_, which is measured at subgroup level in whole population, has indicated lack of equilibrium across the groups and lack of heterozygosity most likely due to the inbreeding nature of rice.

The present study revealed that several unexploited landraces of Tamil Nadu, India which is widely cultivated by the farmers in different parts of the state. Ecological and evolutionary history contributes for the genetic diversity maintained in a population. The varieties with diverse ecosystems and wide eco-geographical conditions contribute for the genetic diversity among rice varieties in this population.

For establishing a core collection for association studies, two step approach followed by Breseghello and Sorrells (2006) and Courtois et al. (2012) was used. This approach involves the determination of population structure and then sampling can be done based on the relatedness of the accessions in the population. Those accessions that show high magnitude of genetic relatedness can be eliminated to develop core collection with diverse representatives. Based on this idea, out of 192 accessions, 150 (Table 5) were selected to form association mapping panel which can be utilized either by genome wide or candidate gene specific association mapping for linking the genotypic and phenotypic variation.

**Table 5:** Genotypes selected for association mapping panel

G. no	Genotypes	G. no	Genotypes	G. no	Genotypes	G. no	Genotypes	G. no	Genotypes	G. no	Genotypes
RG1	Mapillai samba	RG58	Kodaikuluthan	RG113	IG 45(EC 728768- 117736)	RG154	IG 48(EC 729203- 121195)	RG39	Kaatu ponni	RG95	Jeeraga samba
RG2	CK 275	RG59	RPHP 68	RG114	RPHP 159	RG156	IG 12(EC 729626- 121681)	RG41	Godavari samba	RG96	RP-BIO-226
RG3	Senkar	RG60	Rama kuruvaikar	RG115	IG 43(EC 728788- 117759)	RG157	Karungan	RG42	Earapalli samba	RG98	IG 5(EC 729642- 121698)
RG4	Murugankar	RG62	Purple puttu	RG116	RPHP 27	RG158	IG 13(EC 729640- 121696)	RG43	RPHP 129	RG99	IG 31(EC 728844- 117829)
RG5	CHIR 6	RG63	IG 71(EC 728651- 117588)	RG117	IG 65(EC 729024- 120958)	RG159	Sembala	RG44	Mangam samba	RG100	IG 7(EC 729598- 121648)
RG6	CHIR 5	RG65	IG 56(EC 728700- 117658	RG118	Ponmani samba	RG160	IG 72(EC 728650- 117587)	RG45	RPHP 105	RG101	RPHP 52
RG7	Kudai vazhai	RG66	Jeevan samba	RG120	Thattan samba	RG161	Panamarasamba	RG46	IG 4(EC 729639- 121695)	RG102	Varakkal
RG8	CHIR 8	RG67	RPHP 106	RG121	IG 74(EC 728622- 117517)	RG162	IR 64	RG48	Kalarkar	RG103	Mattaikar
RG9	Kuruvai kalanjiyam	RG68	IG 63(EC 728711- 117674)	RG122	Kaliyan samba	RG163	Mikuruvai	RG50	Sornavari	RG104	IG 53(EC 728752- 117719)
RG12	Vellai chithiraikar	RG69	RPHP 48	RG123	IG 2(EC 729808-121874)	RG164	Thillainayagam	RG51	RPHP 134	RG105	IG 6(EC 729592- 121642)
RG14	Jothi	RG70	Karthi samba	RG124	IG 29(EC 728925- 117920)	RG165	ARB 64	RG52	ARB 58	RG106	Katta samba
RG15	Palkachaka	RG71	IG 27(IC 0590934- 121255)	RG126	Kallimadayan	RG166	RPHP 140	RG53	IR 68144-2B-2-2-3-1-127	RG107	RH2-SM-1-2-1
RG17	Chivapu chithiraikar	RG72	Aarkadu kichili	RG127	IG 10(EC 729686- 121743)	RG168	Haladichudi	RG54	PTB 19	RG108	Red sirumani
RG18	CHIR 11	RG74	ARB 65	RG128	IG 75(EC 728587- 117420)	RG169	IG 24(EC 728751- 117718)	RG55	IG 67(EC 729050- 120988)	RG109	Vadivel
RG20	Kalvalai	RG76	Matta kuruvai	RG129	IG 38(EC 728742 - 117707)	RG170	RPHP 42	RG56	RPHP 59	RG110	Norungan
RG22	IR 36	RG77	Karuthakar	RG130	IG 39(EC 728779- 117750)	RG172	IG 25(EC 729728- 121785)	RG57	RPHP 103	RG112	IG 35(EC 728858- 117843)
RG25	Sorna kuruvai	RG80	IG 66(EC 729047- 120985)	RG131	RPHP 90	RG173	IG 73(EC 728627- 117527)	RG143	IG 46(IC 471826- 117647)	RG184	IG 18(EC 728892- 117880)
RG26	Rascadam	RG81	CB-07-701-252	RG132	IG 33(EC 728938- 117935)	RG174	IG 51(EC 728772- 117742)	RG145	IG 60(EC 728730- 117695)	RG185	RPHP 36
RG31	Chinthamani	RG82	Thooyamalli	RG133	IG 42(EC 728798- 117774)	RG175	Vellai kudaivazhai	RG146	IR 75862-206	RG186	IG 28(EC 728920- 117914)
RG32	Thogai samba	RG83	RPHP 93	RG134	IG 9(EC 729682- 121739)	RG176	Kodai	RG147	IG 58(EC 728725- 117689)	RG187	Vadakathi samba
RG33	Malayalathan samba	RG85	RPHP 104	RG135	RPHP 161	RG178	IG 17(EC 728900- 117889)	RG148	Chinna aduku nel	RG188	RPHP 80
RG34	RPHP 125	RG86	RPHP 102	RG136	IG 8(EC 729601- 121651)	RG180	IG 59(EC 728729- 117694)	RG149	RH2-SM-2-23	RG189	IG 41(EC 728800- 117776)
RG35	CK 143	RG89	IR 83294-66-2-2-3-2	RG137	IG 37(EC 728715- 117678)	RG181	IG 52(EC 728756- 117723)	RG150	IG 14(IC 517381- 121422)	RG190	IG 26(IC 0590943- 121899)
RG36	Kattikar	RG91	IG 23(EC 729391- 121419)	RG141	IG 44(EC 728762- 117729)	RG182	ARB 59	RG151	IG 32(EC 728838- 117823)	RG191	IG 15(EC 728910- 117901)
RG37	Shenmolagai	RG92	IG 49(EC 729102- 121052)	RG142	Sasyasree	RG183	RPHP 163	RG152	RPHP 47	RG192	Nootri pathu

## Conclusion

This study analyze the pattern of divergence exists in a population of 192 rice accessions that constitute our rice diversity panel for association mapping. Based on various statistical methods, we identified two sub groups within 192 rice accessions selected for establishing association mapping panel. The average number of alleles per locus and gene diversity has indicated the existence of broad genetic base in this collection. The result of structure analysis is in accordance with clustering method of neighbor joining tree and principal coordinate analysis. Thus, the results of this study which indicates the genetic diversity of the accessions can be utilized to predict approaches such as association analysis, classical mapping population development; parental line selection in breeding programs and hybrid development for exploiting the natural genetic variation exists in this population.

## Methods

### Plant Material

A collection consisting of 192 rice accessions was used in this study, which consist of land races and varieties collected from nine different states of India as well as from Argentina, Bangladesh, Brazil, Bulgaria, China, Colombia, Indonesia, Philippines, Taiwan, Uruguay, Venezuela and United States (Table 6).

**Table 6:** Germplasm accessions used in the study

G. no.	Genotype	Parentage	Origin	Type – traditional/Improved	Subtype	Ecosystem IR = irrigated, RL = rainfed lowland; UP = upland	Maturity class: E = early, M = medium, L = late;	Donors/Original providing country
RG1	Mapillai samba	Landrace	Tamil Nadu, India	T	*indica*	IR	L	India
RG2	CK 275	CO50 X KAVUNI	Tamil Nadu, India	I	*indica*	IR	L	India
RG3	Senkar	Landrace	Tamil Nadu, India	T	*indica*	IR	M	India
RG4	Murugankar	Landrace	Tamil Nadu, India	T	*indica*	UP	L	India
RG5	CHIR 6	Improved chinsurah	West Bengal	I	*indica*	IR	E	India
RG6	CHIR 5	Improved chinsurah	West Bengal	I	*indica*	IR	E	India
RG7	Kudai vazhai	Landrace	Tamil Nadu, India	T	*indica*	UP	E	India
RG8	CHIR 8	Improved chinsurah	West Bengal	I	*indica*	IR	E	India
RG9	Kuruvai kalanjiyam	Landrace	Tamil Nadu, India	T	*indica*	IR	E	India
RG10	Nava konmani	Landrace	Tamil Nadu, India	T	*indica*	RL	M	India
RG11	CHIR 10	Improved chinsurah	West Bengal	I	*indica*	IR	M	India
RG12	Vellai chithiraikar	Landrace	Tamil Nadu, India	T	*indica*	RL	E	India
RG13	CHIR 2	Improved chinsurah	West Bengal	I	*indica*	IR	M	India
RG14	Jyothi	Variety	Kerala, India	T	*indica*	IR	E	India
RG15	Palkachaka	Landrace	Tamil Nadu, India	T	*indica*	IR	M	India
RG16	Thooyala	Landrace	Tamil Nadu, India	T	*indica*	IR	E	India
RG17	Chivapu chithiraikar	Landrace	Tamil Nadu, India	T	*indica*	RL	E	India
RG18	CHIR 11	Improved chinsurah	West Bengal	I	*indica*	IR	M	India
RG19	Koolavalai	Landrace	Tamil Nadu, India	T	*indica*	RL	M	India
RG20	Kalvalai	Landrace	Tamil Nadu, India	T	*indica*	RL	E	India
RG21	Mohini samba	Landrace	Tamil Nadu, India	T	*indica*	IR	M	India
RG22	IR 36	IR 1561 X IR 24 X Oryza nivara x CR 94	IRRI, Philippines	I	*indica*	IR	E	Philippines
RG23	Koombalai	Landrace	Tamil Nadu, India	T	*indica*	IR	M	India
RG24	Tadukan	Landrace	Philippines	T	*indica*	UP	M	Philippines
RG25	Sorna kuruvai	Landrace	Tamil Nadu, India	T	*indica*	IR	M	India
RG26	Rascadam	Landrace	Tamil Nadu, India	T	*indica*	IR	M	India
RG27	Muzhi karuppan	Landrace	Tamil Nadu, India	T	*indica*	IR	E	India
RG28	Kaatukuthalam	Landrace	Tamil Nadu, India	T	*indica*	RL	M	India
RG29	Vellaikattai	Landrace	Tamil Nadu, India	T	*indica*	RL	M	India
RG30	Poongar	Landrace	Tamil Nadu, India	T	*indica*	RL	L	India
RG31	Chinthamani	Landrace	Tamil Nadu, India	T	*indica*	RL	M	India
RG32	Thogai samba	Landrace	Tamil Nadu, India	T	*indica*	RL	M	India
RG33	Malayalathan samba	Landrace	Tamil Nadu, India	T	*indica*	IR	E	India
RG34	RPHP125	NDR 2026 (RICHA)	UTTAR PRADHESH	I	*indica*	IR	E	India
RG35	CK 143	CO50 X KAVUNI	Tamil Nadu, India	I	*indica*	IR	L	India
RG36	Kattikar	Landrace	Tamil Nadu, India	T	*indica*	RL	M	India
RG37	Shenmolagai	Landrace	Tamil Nadu, India	T	*indica*	IR	M	India
RG38	Velli samba	Landrace	Tamil Nadu, India	T	*indica*	IR	M	India
RG39	Kaatu ponni	Landrace	Tamil Nadu, India	T	*indica*	IR	M	India
RG40	kakarathan	Landrace	Tamil Nadu, India	T	*indica*	IR	M	India
RG41	Godavari samba	Landrace	Tamil Nadu, India	T	*indica*	IR	M	India
RG42	Earapalli samba	Landrace	Tamil Nadu, India	T	*indica*	IR	M	India
RG43	RPHP 129	Kamad	JAMMU & KASHMIR	T	*indica*	Scented	E	India
RG44	Mangam samba	Landrace	Tamil Nadu, India	T	*indica*	IR	M	India
RG45	RPHP 105	Moirang phou	MANIPUR	T	*indica*	IR	E	India
RG46	IG 4(EC 729639- 121695)	TD2: :IRGC 9148-1	IRRI, Philippines	I	*indica*	IR	M	Philippines
RG47	Machakantha	Landrace	Orissa, India	T	*indica*	scented	E	India
RG48	Kalarkar	Landrace	Tamil Nadu, India	T	*indica*	RL	E	India
RG49	Valanchennai	Landrace	Tamil Nadu, India	T	*indica*	RL	E	India
RG50	Sornavari	Landrace	Tamil Nadu, India	T	*indica*	RL	E	India
RG51	RPHP 134	NJAVARA	Kerala	T	*indica*	RL	E	India
RG52	ARB 58	Variety	Karnataka	I	*indica*	IR	E	India
RG53	IR 68144-2B-2-2-3-1-127	IR 72 X ZAWA BONDAY	IRRI, Philippines	I	*indica*		E	Philippines
RG54	PTB 19	Variety	Kerala, India	I	*indica*	IR	M	India
RG55	IG 67(EC 729050- 120988)	IR 77384-12-35-3-12-l-B::IRGC 117299-1	IRRI, Philippines	I	*indica*	IR	E	Philippines
RG56	RPHP 59	Taroari Basmati/karnal local	HARYANA	T	*Aromatic*	scented	L	India
RG57	RPHP 103	Pant sugandh dhan -17	UTTARKHAND	I	*Aromatic*	scented	L	India
RG58	Kodaikuluthan	Landrace	Tamil Nadu, India	T	*indica*	RL	E	India
RG59	RPHP 68	Subhdra	Orissa, India	I	*indica*	RL	E	India
RG60	Rama kuruvaikar	Landrace	Tamil Nadu, India	T	*indica*	IR	E	India
RG61	Kallundai	Landrace	Tamil Nadu, India	T	*indica*	RL	E	India
RG62	Purple puttu	Landrace	Tamil Nadu, India	T	*indica*	IR	E	India
RG63	IG 71(EC 728651- 117588)	TEPI BORO::IRGC 27519-1	IRRI, Philippines	I	*aus*	IR	E	Philippines
RG64	Ottadaiyan	Landrace	Tamil Nadu, India	T	*indica*	RL	M	India
RG65	IG 56(EC 728700- 117658	BICO BRANCO	Brazil	T	*Aromatic*	UP	E	Philippines
RG66	Jeevan samba	Landrace	Tamil Nadu, India	T	*indica*	IR	M	India
RG67	RPHP 106	akut phou	MANIPUR	I	*indica*	IR	M	India
RG68	IG 63(EC 728711- 117674)	CAAWA/FORTUNA	IRRI, Philippines	I	*Tropical Japonica*	IR	M	Philippines
RG69	RPHP 48	Bindli	UTTARKHAND	T	*Aromatic*	Scented	L	India
RG70	Karthi samba	Landrace	Tamil Nadu, India	T	*indica*	IR	M	India
RG71	IG 27(IC 0590934- 121255)	ARC 11345::IRGC 21336-1	IRRI, Philippines	I	*indica*	IR	M	Philippines
RG72	Aarkadu kichili	Landrace	Tamil Nadu, India	T	*indica*	IR	M	India
RG73	Kunthali	Landrace	Tamil Nadu, India	T	*indica*	IR	E	India
RG74	ARB 65	Variety	Karnataka	I	*indica*	IR	E	India
RG75	IG 21(EC 729334- 121355)	HONGJEONG::IRGC 73052-1	IRRI, Philippines	I	*japonica*	IR	E	Philippines
RG76	Matta kuruvai	Landrace	Tamil Nadu, India	T	*indica*	IR	E	India
RG77	Karuthakar	Landrace	Tamil Nadu, India	T	*indica*	RL	E	India
RG78	RPHP 165	Tilak kachari	West Bengal	T	*indica*	IR	E	India
RG79	Manavari	Landrace	Tamil Nadu, India	T	*indica*	U	E	India
RG80	IG 66(EC 729047- 120985)	IR 71137-243-2-2-3-3::IRGC 99696-1	IRRI, Philippines	I	*indica*	IR	E	Philippines
RG81	CB-07-701-252	White ponni X Rasi	Tamil Nadu, India	I	*indica*	IR	E	India
RG82	Thooyamalli	Landrace	Tamil Nadu, India	T	*indica*	IR	M	India
RG83	RPHP 93	Type-3 (Dehradooni Basmati)	UTTARKHAND	I	*indica*	Scented	M	India
RG84	Velsamba	Landrace	Tamil Nadu, India	T	*indica*	IR	M	India
RG85	RPHP 104	Kasturi (IET 8580)	UTTARKHAND	I	*indica*	IR	M	India
RG86	RPHP 102	Kanchana	Kerala, India	I	*indica*	Semi Deep Water	L	India
RG87	IG 40(EC 728740- 117705)	DEE GEO WOO GEN	TAIWAN	T	*Indica*	IR	M	Philippines
RG88	Saranga	Landrace	Tamil Nadu, India	T	*indica*	IR	E	India
RG89	IR 83294-66-2-2-3-2	DAESANBYEO X IR65564-44-5-1	IRRI, Philippines	I	*japonica*	RL	M	Philippines
RG90	IG 61(EC 728731- 117696)	CRIOLLO LA FRIA	Venezuela	I	*Indica*	IR	E	Philippines
RG91	IG 23(EC 729391- 121419)	MAHA PANNITHI::IRGC 51021-1	IRRI, Philippines	I	*Aus*	IR	M	Philippines
RG92	IG 49(EC 729102- 121052)	MENAKELY ::IRGC 69963-1	Madagascar	I	*Indica*	RL	M	Philippines
RG93	Uppumolagai	Landrace	Tamil Nadu, India	T	*Indica*	IR	M	India
RG94	Karthigai samba	Landrace	Tamil Nadu, India	T	*Indica*	RL	M	India
RG95	Jeeraga samba	Landrace	Tamil Nadu, India	T	*Indica*	IR	M	India
RG96	RP-BIO-226	IMPROVED SAMBHA MAHSURI	ANDHRA PRADESH	I	*Indica*	IR	M	India
RG97	Varigarudan samba	Landrace	Tamil Nadu, India	T	*Indica*	IR	M	India
RG98	IG 5(EC 729642- 121698)	IR 65907-116-1-B::C1	IRRI, Philippines	I	*japonica*	UP	E	Philippines
RG99	IG 31(EC 728844- 117829)	ORYZICA LLANOS 5	Colombia	T	*Indica*	IR	M	Philippines
RG100	IG 7(EC 729598- 121648)	VARY MAINTY::IRGC 69910-1	Madagascar	I	*japonica*	IR	M	Philippines
RG101	RPHP 52	SEBATI	Orissa, India	I	*Indica*	IR	M	India
RG102	Varakkal	Landrace	Tamil Nadu, India	T	*Indica*	UP	E	India
RG103	Mattaikar	Landrace	Tamil Nadu, India	T	*Indica*	RL	L	India
RG104	IG 53(EC 728752- 117719)	CAROLINA RINALDO BARSANI	URUGUAY	I	*Temperate japonica*	IR	E	Philippines
RG105	IG 6(EC 729592- 121642)	SOM CAU 70 A::IRGC 8227-1	Vietnam	I	*Temperate japonica*	IR	E	Philippines
RG106	Katta samba	Landrace	Tamil Nadu, India	T	*Indica*	RL	L	India
RG107	RH2-SM-1-2-1	SWARNA X MOROBERAKAN	Tamil Nadu, India	I	*Indica*	IR	E	India
RG108	Red sirumani	Landrace	Tamil Nadu, India	T	*Indica*	RL	E	India
RG109	Vadivel	Landrace	Tamil Nadu, India	T	*Indica*	IR	M	India
RG110	Norungan	Landrace	Tamil Nadu, India	T	*Indica*	RL	E	India
RG111	IG 20(EC 729293- 121310)	CHIGYUNGDO::IRGC 55466-1	South Korea	I	*Indica*	UP	E	Philippines
RG112	IG 35(EC 728858- 117843)	PATE BLANC MN 1	Cote D’Ivoire	I	*japonica*	UP	M	Philippines
RG113	IG 45(EC 728768- 117736)	FORTUNA	Puerto Rico	T	*japonica*	IR	M	Philippines
RG114	RPHP 159	Radhuni Pagal	BANGLADESH	I	*aromatic rice*	Scented	L	India
RG115	IG 43(EC 728788- 117759)	IR-44595	IRRI, Philippines	I	*indica*	IR	E	Philippines
RG116	RPHP 27	Azucena	IRRI, Philippines	T	*Tropical Japonica*	RL	E	India
RG117	IG 65(EC 729024- 120958)	GODA HEENATI::IRGC 31393-1	SRILANKA	I	*indica*	IR	E	Philippines
RG118	Ponmani samba	Landrace	Tamil Nadu, India	T	*indica*	IR	M	India
RG119	Ganthasala	Landrace	Tamil Nadu, India	T	*indica*	IR	M	India
RG120	Thattan samba	Landrace	Tamil Nadu, India	T	*indica*	IR	E	India
RG121	IG 74(EC 728622- 117517)	KINANDANG PATONG::IRGC 23364-1	IRRI, Philippines	I	*japonica*	RL	M	Philippines
RG122	Kaliyan samba	Landrace	Tamil Nadu, India	T	*indica*	IR	M	India
RG123	IG 2(EC 729808-121874)	BLUEBONNET 50::IRGC 1811-1	IRRI, Philippines	I	*japonica*	UP	M	Philippines
RG124	IG 29(EC 728925- 117920)	TOX 782-20-1	NIGERIA	T	*Tropical Japonica*	IR	E	Philippines
RG125	RPHP 55	Kalinga -3	Orissa	I	*indica*	RL	E	India
RG126	Kallimadayan	Landrace	Tamil Nadu, India	T	*indica*	RL	E	India
RG127	IG 10(EC 729686- 121743)	HASAN SERAI	IRRI, Philippines	I	*aromatic*	IR	E	Philippines
RG128	IG 75(EC 728587- 117420)	AEDAL::IRGC 55441-1	Korea	T	*japonica*	IR	E	Philippines
RG129	IG 38(EC 728742 - 117707)	DELREX	UNITED STATES		*Tropical japonica*	IR	M	Philippines
RG130	IG 39(EC 728779- 117750)	HONDURAS	HONDURAS		*indica*	IR	M	Philippines
RG131	RPHP 90	182(M)	Andhra Pradesh	I	*indica*	IR	E	India
RG132	IG 33(EC 728938- 117935)	WC 3397	JAMAICA		*Tropical Japonica*	IR	E	Philippines
RG133	IG 42(EC 728798- 117774)	KALUBALA VEE	SRILANKA	T	*indica*	IR	E	Philippines
RG134	IG 9(EC 729682- 121739)	GEMJYA JYANAM::IRGC 32411-C1	IRRI, Philippines	I	*indica*	IR	E	Philippines
RG135	RPHP 161	Champa Khushi	Vietnam	T	*indica*	UP	E	India
RG136	IG 8(EC 729601- 121651)	XI YOU ZHAN::IRGC 78574-1	China	I	*indica*	IR	E	Philippines
RG137	IG 37(EC 728715- 117678)	CENIT	ARGENTINA	T	*Tropical Japonica*	IR	L	Philippines
RG138	Sigappu kuruvikar	Landrace	Tamil Nadu, India	T	*indica*	RL	E	India
RG139	RPHP 138	EDAVANKUDI POKKALI	Kerala, India	T	*indica*	Deep water	L	India
RG140	Raja mannar	Landrace	Tamil Nadu, India	T	*indica*	IR	M	India
RG141	IG 44(EC 728762- 117729)	EDITH	UNITED STATES	T	*indica*	IR	E	Philippines
RG142	Sasyasree	TKM 6 x IR 8	West Bengal	I	*indica*	IR	E	India
RG143	IG 46(IC 471826- 117647)	BABER	INDIA	I	*indica*	IR	E	India
RG144	Chetty samba	Landrace	Tamil Nadu, India	T	*indica*	IR	E	India
RG145	IG 60(EC 728730- 117695)	CREOLE	Belize	T	*indica*	IR	M	Philippines
RG146	IR 75862-206	IR 75083 X IR 65600 -81-5-3-2	IRRI, Philippines	I	*Tropical Japonica*	IR	M	Philippines
RG147	IG 58(EC 728725- 117689)	CI 11011	UNITED STATES		*japonica*	IR	M	Philippines
RG148	Chinna aduku nel	Landrace	Tamil Nadu, India	T	*indica*	IR	L	India
RG149	RH2-SM-2-23	SWARNA X MOROBERAKAN	Tamil Nadu, India	I	*indica*	IR	M	India
RG150	IG 14(IC 517381- 121422)	MALACHAN::IRGC 54748-1	India	I	*indica*	UP	E	Philippines
RG151	IG 32(EC 728838- 117823)	NOVA	United States	I	*japonica*	IR	M	Philippines
RG152	RPHP 47	Pathara (CO-18 x Hema)	India	I	*indica*	IR	E	India
RG153	Sembilipiriyan	Landrace	Tamil Nadu, India	T	*indica*	RL	M	India
RG154	IG 48(EC 729203- 121195)	DINOLORES::IRGC 67431-1	IRRI, Philippines	I	*indica*	UP	M	Philippines
RG155	Sona mahsuri	Landrace	Tamil Nadu, India	T	*indica*	IR	E	India
RG156	IG 12(EC 729626- 121681)	SHESTAK::IRGC 32351-1	Iran	I	*indica*	IR	E	Philippines
RG157	Karungan	Landrace	Tamil Nadu, India	T	*indica*	IR	E	India
RG158	IG 13(EC 729640- 121696)	CURINCA::C1	BRAZIL	I	*indica*	IR	E	Philippines
RG159	Sembala	Landrace	Tamil Nadu, India	T	*indica*	IR	L	India
RG160	IG 72(EC 728650- 117587)	TD 25::IRGC 9146-1	Thailand	I	*indica*	IR	M	Philippines
RG161	Panamarasamba	Landrace	Tamil Nadu, India	T	*indica*	IR	M	India
RG162	IR 64	IR-5857-33-2-1 x IR-2061-465-1-5-5	IRRI, Philippines	I	*indica*	IR	E	Philippines
RG163	Mikuruvai	Landrace	Tamil Nadu, India	T	*indica*	RL	E	India
RG164	Thillainayagam	Landrace	Tamil Nadu, India	T	*indica*	IR	M	India
RG165	ARB 64	Variety	Karnataka	I	*indica*	IR	E	India
RG166	RPHP 140	VYTILLA ANAKOPON	Kerala	T	*indica*	IR	E	India
RG167	IG 70(EC 729045- 120983)	IR43::IRGC 117005-1	IRRI, Philippines	I	*indica*	IR	M	Philippines
RG168	Haladichudi	Landrace	Orissa, India	T	*indica*	IR	E	India
RG169	IG 24(EC 728751- 117718)	DNJ 140	BANGLADESH	I	*Aus*	IR	E	Philippines
RG170	RPHP 42	Salimar Rice -1	JAMMU & KASHMIR	I	*indica*	IR	M	India
RG171	RPHP 44	BR- 2655	KARNATAKA	I	*indica*	IR	L	India
RG172	IG 25(EC 729728- 121785)	LOHAMBITRO 224::GERVEX 5144-C1	Madagascar	I	*Tropical Japonica*	IR	E	Philippines
RG173	IG 73(EC 728627- 117527)	MAKALIOKA 34::IRGC 6087-1	IRRI, Philippines	I	*indica*	IR	E	Philippines
RG174	IG 51(EC 728772- 117742)	GOGO LEMPUK	Indonesia		*Tropical Japonica*	IR	M	Philippines
RG175	Vellai kudaivazhai	Landrace	Tamil Nadu, India	T	*indica*	RL	M	India
RG176	Kodai	Landrace	Tamil Nadu, India	T	*indica*	RL	E	India
RG177	Kallundaikar	Landrace	Tamil Nadu, India	T	*indica*	UP	M	India
RG178	IG 17(EC 728900- 117889)	SIGADIS	INDONESIA	T	*indica*	RL	L	Philippines
RG179	Avasara samba	Landrace	Tamil Nadu, India	T	*indica*	IR	E	India
RG180	IG 59(EC 728729- 117694)	COPPOCINA	BULGARIA	I	*Tropical Japonica*	IR	M	Philippines
RG181	IG 52(EC 728756- 117723)	DOURADO AGULHA	BRAZIL	I	*Tropical Japonica*	IR	M	Philippines
RG182	ARB 59	Variety	Karnataka	I	*indica*	IR	E	India
RG183	RPHP 163	Seeta sail	West Bengal	T	*indica*	Scented	M	India
RG184	IG 18(EC 728892- 117880)	SERATOES HARI	INDONESIA	T	*indica*	IR	E	Philippines
RG185	RPHP 36	TKM-9	Tamil Nadu, India	I	*indica*	IR	E	India
RG186	IG 28(EC 728920- 117914)	TIA BURA	INDONESIA	T	*Tropical Japonica*	IR	M	Philippines
RG187	Vadakathi samba	Landrace	Tamil Nadu, India	T	*indica*	IR	M	India
RG188	RPHP 80	24(K)	Andhra Pradesh	I	*indica*	IR	E	India
RG189	IG 41(EC 728800- 117776)	KANIRANGA	Indonesia	T	*Tropical japonica*	IR	M	Philippines
RG190	IG 26(IC 0590943- 121899)	BASMATI 370::IRGC 3750-1	IRRI, Philippines	I	*aromatic*	IR	E	Philippines
RG191	IG 15(EC 728910- 117901)	SZE GUEN ZIM	CHINA	I	*indica*	IR	E	Philippines
RG192	Nootri pathu	Landrace	Tamil Nadu, India	T	*indica*	RL	L	India

IRRI lines - The number after hyphen inside brackets represent IRGC number

### Microsatellite Genotyping

#### DNA Isolation and PCR Amplification

DNA was extracted from leaf tissue by grinding with liquid nitrogen using CTAB method (Saghai-Maroof et al. 1984.). It was diluted to a final concentration of 30 ng μl^−1^ for enabling polymerase chain reactions. DNA amplification parameters such as specificity, efficiency and fidelity are strongly influenced by the components of the PCR reaction and by thermal cycling conditions (Caetano-Anolles and Brant 1991). Therefore, the careful optimization of reaction components and conditions will ultimately result in more reproducible and efficient amplification. The concentrations of primers, template DNA, Master Mix, and annealing temperature was optimized on eight diverse accessions for 156 SSR markers distributed on the 12 chromosomes by modified Taguchi method (Cobb and CIarkson 1994). Microsatellite primer sequences, annealing temperature and chromosomal locations are obtained from GRAMENE database (http://archive.gramene.org/markers/microsat/). Sixty one SSR primer pairs which produce polymorphic allele amplification were chosen to genotype the entire set of germplasm collection.

The volume of the PCR reaction system was 10 μl. The PCR reaction mixture of 10 μl had 0.4 mM dNTPs, 4 mM of MgCl_2_, 150 mM of Tris–HCl, 10 pmoles of forward and reverse primer and 0.05 U *Taq* polymerase with 30 ng of DNA. Polymerase chain reaction was performed in BIORAD THERMAL CYCLER using the following program: 94 °C for 2 min, 35 cycles of 94 °C for 45 sec, 50–60 °C for 1 min, 72 °C for 2 min with a final extension of 72 °C for ten min.

### Polyacrylamide Gel Electrophoresis

Amplified products were size separated in native polyacrylamide gel electrophoresis using 6 % (*w/v*) polyacrylamide gel according to Sambrook et al. (2001) in vertical electrophoresis tank with 1X TBE at 150 V. The gel size was determined using standard molecular weight size markers after the bands were detected by silver staining.

### Allele Scoring

The bands were visualized in a cluster of two to six in the stained gels for most of the markers. Based on the expected product size given in the GRAMENE website (Additional file 2: Table S1), the size of the most intensely amplified bands around the expected product size for each microsatellite marker was identified using standard molecular weight size markers (20 bp DNA ladder, GeNeI Company). Then the stained gel was dried and documented using light box. Allele score was given based on the presence of a particular size allele in each of the germplasm. The presence was denoted as 1 and absence of an allele as 0 and it was rechecked manually (Additional file 3: Table S2).

### Data Analysis

A 1/0 matrix was constructed based on the presence and absence of alleles for the set of 61 markers. This SSR genotype data was analyzed for genetic diversity and population structure.

### Genetic Diversity

For a set of accessions, genetic diversity parameters such as number of alleles per locus, allele frequency, heterozygosity and polymorphic information index (PIC) was estimated using the program POWERMARKER Ver3.25 (Liu and Muse 2005). Allele frequency represents the frequency of particular allele for each marker. Heterozygosity is the proportion of heterozygous individuals in the population. Polymorphic information content that represent the amount of polymorphism within a population was estimated based on Botstein et al. (1980).

To assess genetic structure, model based approach and distance based approach were used. Model based approach was utilized with Structure ver 2.3.4 software (Pritchard et al. 2000). The actual number of subpopulation which is denoted by K was identified by this method. For that, the project was run with the following parameter set: the possibility of admixture and allele frequency correlated. Run length was given as 150,000 burning period length followed by 150,000 Markov Chain Monte Carlo (MCMC) replication. Each k value was run for 10 times with k value varying from 1 to 10. The optimum k value was determined by plotting the mean estimate of the log posterior probability of the data (L (K) against the given K value. True number of subpopulation was identified using the maximal value of L (K). An adhoc quantity ΔK proposed by (Evanno et al. 2005) based on second order rate of change of the likelihood function with respect to K estimated using Structure Harvester (Earl 2012) has also shown a clear peak at the optimal K value.

Distance based approach which is based on calculating pair wise distance matrix was computed by calculating a dissimilarity matrix using a shared allele index with DARwin software (Perrier and Jacquemoud-Collet 2006). An unweighted neighbor joining tree was constructed using the calculated dissimilarity index. The genetic distance between accessions was estimated using NEI coefficient (Nei 1972) with bootstrap procedure of resampling (1000) across markers and individuals from allele frequencies. To determine the association among the accessions, unweighted pair group method with arithmetic mean (UPGMA) tree was also drawn using Powermarker and viewed in MEGA 6.0 software (Tamura et al. 2013).

The presence of molecular variance within and between hierarchical population structure estimated by Structure was assessed via Analysis of molecular variance (AMOVA) by Arlequin (Excoffier et al. 2005). F statistics which include F_IT,_ deviations from Hardy- Weinberg expectation across the whole population, F_IS_ deviation from Hardy- Weinberg expectation within a population and F_ST,_ correlation of alleles between subpopulation was calculated using AMOVA approach in Arlequin. AMOVA and Principal Coordinate analysis of the germplasm set was performed based on Nei (Nei 1973) distance matrix using GenAlEx 6.5 (Peakall and Smouse 2012).

## Additional files

Additional file 1: Figure S1. Allelic pattern of different SSR markers used in this study. (JPG 1.03 MB) Additional file 2: Table S1. Expected product size obtained from Gramene and observed product size for the SSR markers used in this study. (XLS 170 kb) Additional file 3: Table S2. Allele matrix of 192 accessions x 61 SSRs. (XLSX 10 kb)
