# Supplementary figures and images for: Analysis of Population Structure and Genetic Diversity in Rice Germplasm Using SSR Markers: An Initiative Towards Association Mapping of Agronomic Traits in Oryza Sativa
Source: Rice (N Y). 2015 Sep 26;8:30. doi: 10.1186/s12284-015-0062-5 (PMC4583558; doi:10.1186/s12284-015-0062-5)

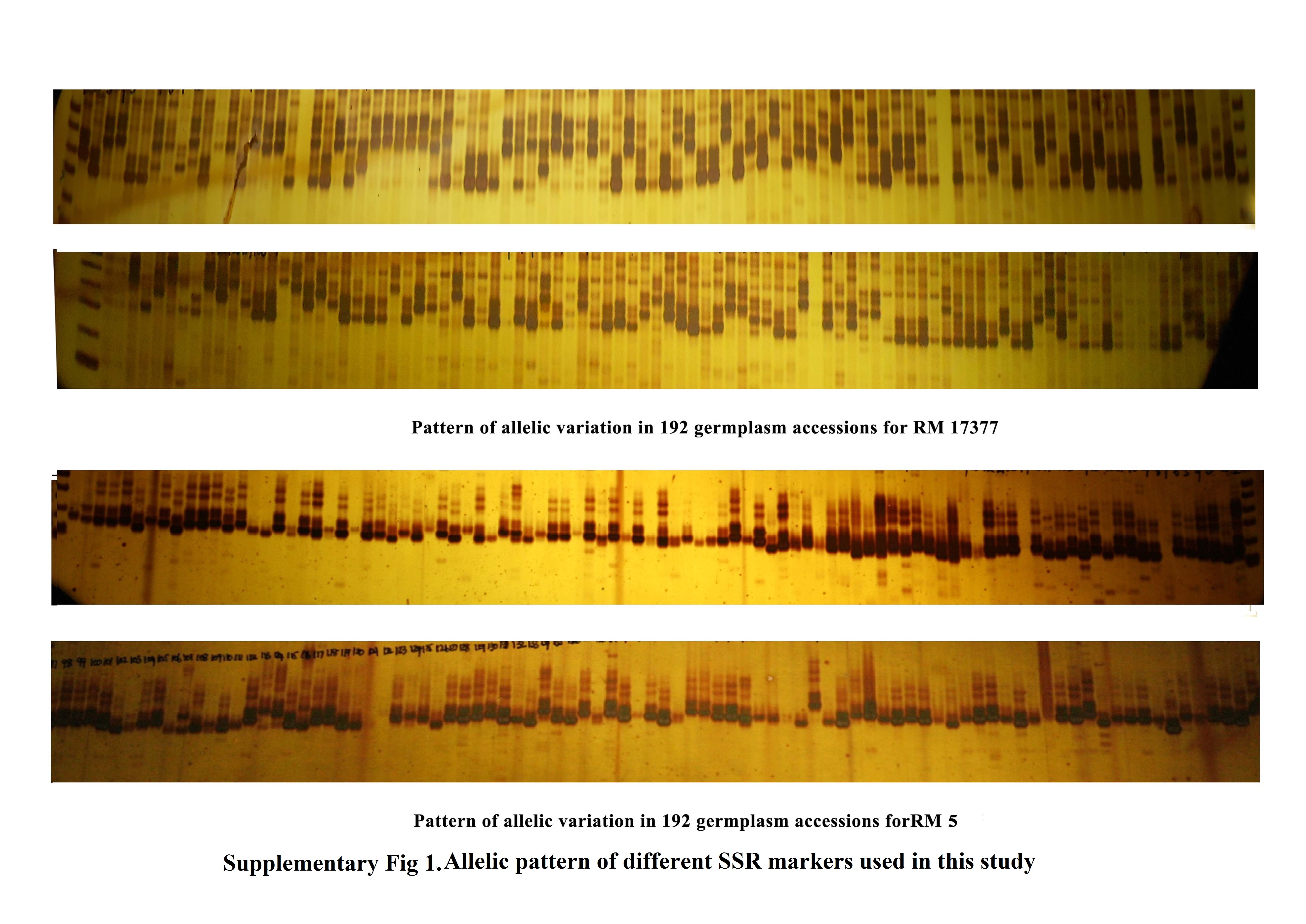

Supplement: Additional file 1: Figure S1. — Allelic pattern of different SSR markers used in this study. (JPG 1.03 MB) [file 12284_2015_62_MOESM1_ESM.jpg]
